# Utilisation of the National Antiretroviral Therapy Guidelines among health care professionals working in Abuja treatment centres, Nigeria

**DOI:** 10.4102/phcfm.v1i1.27

**Published:** 2009-07-21

**Authors:** Lindiwe I. Zungu, Ugbede O. Abu, Gboyega A. Ogunbanjo, Geoffrey K. Setswe

**Affiliations:** 1 Department of Environmental Occupational Health, University of Limpopo, South Africa; 2 Institute of Human Virology, Nigeria; 3 Department of Family Medicine, University of Limpopo, South Africa; 4 Human Sciences Research Council, South Africa

**Keywords:** accessibility, utilisation, National Antiretroviral Treatment Guidelines, health care professionals, Nigeria

## Abstract

**Background:**

Access to and utilisations of the National Antiretroviral Treatment Guidelines (NATG) are valuable factors for effective programme implementation. The objective of this study was to investigate the accessibility of the NATG and their utilisation by health care professionals from five treatment centres in Abuja, Nigeria.

**Method:**

A quantitative cross-sectional descriptive survey was conducted in 2007 using purposively sampled health care professionals. Questionnaires were self-administered to participants who consented in writing to participate in the survey.

**Results:**

97 health care professionals participated in this study with about equal numbers of men and women: 48 (49.5%) women and 49 (50.5%) men. Of these, 21.6% were unaware of the existence of the NATG in their treatment centres. More than half (51.5%) reported that they did not have access to the NATG as opposed to those (48.5%) who had access to the guidelines. Furthermore, 16.5% of the participants confirmed that they had access to an institutional copy of the NATG while 14.4% indicated that they had individual copies and only 3.1% stated that they had individual copies and access to the hospital copy as well. Regarding utilisation of the NATG, 41.2% rarely used them, 32.9% never used them and only 25.7% often used them. The most frequent use of the NATG was among pharmacists (38.1%) compared to the least frequent use among nurses (20.0%).

**Conclusion:**

Poor accessibility of the NATG may have a negative impact on guidelines utilisation among health care professionals in Nigeria.

## INTRODUCTION AND BACKGROUND

Disease-specific initiatives for the management of particular diseases encourage adherence to evidence-based guidelines and ensure that such adherence is monitored. The goal is to maximise the benefits to patients with specific diseases by encouraging standardisation (i.e. guideline-driven prescription of medication) among providers of health care, which prevents variation of the patterns of practice among health care providers.^1^ Such guidelines are developed by national organisations and provide recommendations, including the use of a multidrug regimen for treatment of patients with a particular disease. In South Africa the National Antiretroviral Therapy Guidelines (NATG) are available to promote optimal therapy and good clinical outcomes for people with HIV/AIDS.^2^ Furthermore, the purpose of the NATG is to set standards as the basis for the use of antiretroviral therapy (ART) drugs upon which training and support programmes should be based.

Similarly, in Nigeria the NATG are intended to serve as a basis for scaling-up and decentralisation of the ART initiative programme management and its implementation at different facility levels. In a bid to improve the quality of care and practice, the World Health Organization (WHO) and the governments of some countries have identified the need to standardise the use of ARVs, which has serious financial implications for world bodies and national governments, coupled with the fatal consequences of the misuse of these drugs. The Federal Government of Nigeria has put in place guidelines for the use of ARV drugs in Nigeria to ensure that quality care is delivered to all people living with HIV/AIDS (PLWHA) as part of the implementation of the National Antiretroviral Access Program.^3^ The guidelines provide essential and relevant information needed by health workers not only to understand HIV/AIDS but also to effectively treat and manage it.

Health care professionals are expected to fully utilise the guidelines to ensure uniformity in the management of HIV/AIDS in Nigeria according to a set standard. The aim of this study was to identify the gaps in relation to the implementation of the NATG (i.e. the distribution and utilisation of the guidelines by health care professionals in Abuja).

## METHOD

A quantitative cross-sectional descriptive survey was conducted in 2007 using purposively sampled health care professionals from five ARV treatment centres in Abuja. The sites selected for the study were those offering comprehensive HIV/AIDS care and treatment to the general public (i.e. secondary, tertiary and faith-based organisations), all located in Abuja. They included the National Hospital Abuja (NHA), Asokoro District Hospital (ADH), Wuse General Hospital (WGH), the University of Abuja Teaching Hospital (UATH) and Saint Mary Catholic Hospital (SMCH). Questionnaires were self-administered by participants at the study sites with the help of a trained research assistant who facilitated the distribution and collection of the questionnaires. Those who consented to participate in the study gave a written informed consent.

Ethical clearance to conduct the study was obtained from the Medunsa Research and Ethics Committee of the University of Limpopo (project number MCREC/PH/88/2007: PG). Approval was also sought from the senior management and research committees of the targeted study sites in Abuja. Confidentiality and anonymity were ensured throughout the execution of the study as participants were requested not to disclose their personal details on the questionnaires. Participants were requested to respond to the questionnaire by using a four-grade Likert scale, with ratings from excellent to poor.

## RESULTS

A total of 98.8% of the sampled participants completed the questionnaires; 48 (49.5%) of the participants were female and 49 (50.5%) were male. The mean age of the female participants was 35.4 years while that of the male participants was 37.4 years. As shown in Table 1, various health care professionals participated in the study: medical doctors (32%), laboratory scientists (24%), pharmacists (22%) and professional nurses (22%). With regard to the number of years working at the Abuja treatment centres among the study participants, 33 (34.0%) had between five and 10 years experience while only 13 (13.4%) had over 20 years work experience in the targeted treatment centres.

**TABLE 1:** HIV/AIDS management training

PROFESSION	NUMBER (%)	

	YES	NO
Doctors	31 (96.9)	1 (3.1)
Lab scientists	12 (54.5)	10 (45.5)
Nurses	21 (100)	-
Pharmacists	20 (90.9)	2 (9.1)

A total of 84 participants (86.5%) indicated that they had received special training in HIV/AIDS management, compared to 13 (13.4%) who had not received any training. The various proportions of participants for each profession who had received training in HIV/AIDS management were doctors 31 (96.9%), nurses 21 (100%), pharmacists 20 (90.9%) and laboratory scientists 12 (54.5%) (see Table 1).

**FIGURE 1:**
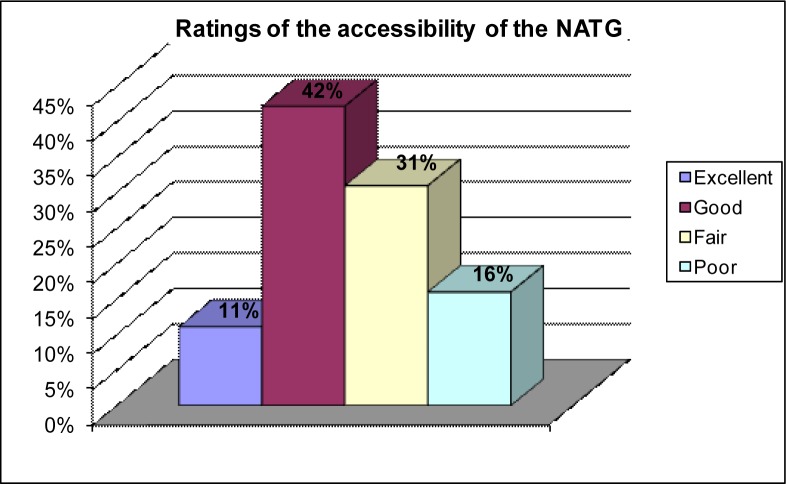
Level of accessibility of the NATG

Regarding awareness of and access to the NATG, the majority of the participants 76 (78.4%) indicated that they were aware of the existence of the NATG in their ART treatment centres while 21 (21.6%) were not aware of the existence of the guidelines. 47 (48.5%) participants were of the opinion that the guidelines were accessible in their centres while 50 (51.5%) reported that the guidelines were not accessible. Participants who had access to the guidelines further rated the extent of accessibility in their centres using a Likert scale of excellent (4), good (3), fair (2) and poor (1). Just over two-fifths or 20 (42.2%) rated the level of accessibility as good followed by 15 (31.1%) who rated it as fair, as depicted in Figure 1.

**FIGURE 2:**
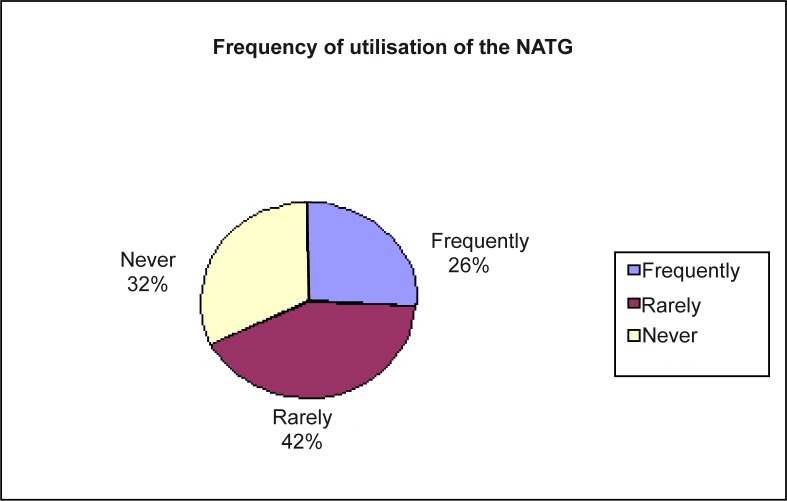
Frequency of utilisation of the NATG

On the frequency of using the NATG, 25 (25.7%) participants reported that they used and/or referred to the guidelines frequently, 40 (41.2%) rarely used them and 32 (32.9%) reported that they never used or referred to the guidelines during patient consultation (see Figure 2).

Furthermore, the study compared the extent of NATG utilisation among the different health care professionals from all the study sites. Participants rated their responses in a ranked order of frequently, rarely and never. The results revealed that eight (25.0%) doctors and eight (38.1%) pharmacists frequently used the NATG guidelines. Similarly, an equal number of five (21.7%) laboratory scientists and five (21.7%) nurses frequently used the guidelines (see Table 2). The results further revealed that doctors had the highest percentage (59.4%) for rarely utilising the guidelines when compared to other health care professionals. The highest percentage (56.5%) for never using the guidelines occurred among the laboratory scientists.

**TABLE 2:** Utilisation of the NATG among the different categories of health care professionals

PROFESSION	FREQUENTLY	RARELY	NEVER	TOTAL
	n (%)	n (%)	n (%)	(n = 97)
Doctors	8 (25.0%)	19 (59.4%)	5 (15.6%)	32
Lab scientists	5 (21.7%)	5 (21.7%)	13 (56.5%)	23
Nurses	5 (21.7%)	8 (40.0%)	8 (40.0%)	21
Pharmacists	8 (38.1%)	8 (38.1%)	5 (23.8%)	21
				
**TOTAL**	**26**	**40**	**31**	**97**

Table 3 shows the different levels of NATG utilisation among the various study sites. The results indicated that the NHA had the highest level – 12 (45.8%) – of frequently utilising the NATG, followed by the ADH with eight (44.4%). Conversely, the UATH had the highest percentages – 13 (48.1%) and 12 (44.4%) – of rarely and never utilising the NATG respectively.

**TABLE 3:** Utilisation of the NATG among the various study sites

ART	FREQUENTLY	RARELY	NEVER	TOTAL
CENTRE	n (%)	n (%)	n (%)	(n = 97)
ADH	8 (44.4%)	4 (22.2%)	6 (33.3%)	18
NHA	12 (45.8%)	10 (41.7%)	3 (12.5%)	25
SMCH	3 (33.3%)	5 (55.6%)	1 (11.1%)	9
UATH	2 (7.4%)	13 (48.1%)	12 (44.4%)	27
WGH	1 (5.5%)	8 (44.4%)	9 (50%)	18
				
**TOTAL**	**26**	**40**	**31**	**97**

## DISCUSSION

The availability and accessibility of treatment guidelines such as the NATG is the key to effective implementation of public health programmes, especially because HIV/AIDS is one of the major public health challenges facing most countries globally. Non-utilisation or improper use of the NATG has serious implications for the quality of service that will reach the PLWHAs who are accessing ARV treatment.

The results show that 97 individuals from various health care professional backgrounds participated in this study from five ARV centres in Abuja that met the inclusion criteria. The findings reveal that there was a fair representation of men and women among the study participants as male participants constituted 50.5% and female participants 49.5% of the study population.

The results further reveal that most of the participants have received ART and/or HIV/AIDS management training but to a different extent. In terms of coverage, the nurses have a wider coverage as all of them (100%) have received training in ART and/ or HIV/AIDS management. On the other hand, a fair number of laboratory scientists (45.5%) have not received training ART and/or HIV/AIDS management training when compared to the other professionals such as doctors and pharmacists.

The results also reveal that 21.6% of health care professionals working at the targeted ART clinics are not aware of the existence of the NATG, although a high proportion (78.4%) of participants are aware of the existence of these guidelines. A similar study on the awareness of clinical practice guidelines among physicians indicated that more than 10% of the physicians were not aware of the existence of such guidelines.^4^ Another related study done in Nigeria among surgery trainees showed that 40% of the participants had an idea about the existence of the CDC guidelines for universal precautions against blood-borne pathogens.^5^, ^6^ The results further revealed that 22.2% knew the contents very well while 17.8% had no idea about the existence of the guidelines. Based on the findings of these studies, it can be deduced that health care professionals have varying degrees of awareness and knowledge of the existence of guidelines for quality and efficient service delivery related to their practices.

In line with the objective of the current study, the accessibility of the NATG among participants was measured by assessing their responses as to whether they had copies of the NATG in their respective institutions. Furthermore, an enquiry was made to find out whether the NATG were accessible to them in their respective consulting rooms/work areas.

The results show that the NATG are not accessible to many of the health care professionals working at the Abuja treatment centres as more than half (51.5%) of the participants indicated that they were not accessible while a smaller proportion (48.5%) indicated that they were accessible. This finding shows that slightly above half of the health care professionals working at the Abuja treatment centres do not have access to the guidelines. In addition, a small percentage (11%) of the participants indicated that the accessibility of the NATG among health care professionals working in the Abuja ART centres was excellent in comparison to those (16%) who declared that it was poor.

Considering the frequency of the use of the NATG among the participants, a good proportion (41.2%) of the participants rarely use the NATG and close to one-third (32.9%) of the participants have never used the NATG. The results further indicate that a small proportion (26.8%) of participants frequently use the NATG compared to those who rarely use the guidelines (41.2%) and those who have never used them (32.9%).

There are thus varying levels of utilisation of the NATG among health care professionals working at the ART centres in Abuja and the reason(s) for such was/were not explored in this study. A related study that reviewed operational challenges encountered by health care workers over the past decade in the global scale-up of ARVs included the non-utilisation of the prescribed NATG due to various reasons, such as poor distribution or dissemination of these guidelines.

This observation is in line with the gap identified with respect to the accessibility of the guidelines because the guidelines can only be put to use when they are available and accessible. The frequency of the use of the NATG is subject to their accessibility; among other related factors this relationship can be examined in subsequent studies related to this area. In South Africa, the National Department of Health has stipulated on the NATG that all health care workers in public and private sectors must familiarise themselves with the contents of the guidelines as a standard method of practice for effective management of HIV/ AIDS.^2^

The results of a similar study identified about seven general categories of barriers to physician adherence to clinical guidelines. Previous studies on the knowledge and utilisation of national treatment guidelines by health care professionals showed that many of them lacked in-depth knowledge of the guidelines; nevertheless, thorough knowledge of these guidelines was observed in some settings.^4^ Various barriers to the utilisation of the NATG have been revealed by previous studies, which include among others lack of knowledge due to lack of awareness of and familiarity with the guidelines.

Other personal factors include a negative attitude or lack of motivation on the part of health care workers, lack of self-efficacy and lack of outcome expectancy. External barriers, guideline factors and environmental factors were identified as factors that can influence behaviour.^4^

A study conducted on adherence to national ART guidelines in Rio de Janeiro, Brazil, showed that physicians at different treatment centres are likely to have different levels of knowledge and application with regard to the NATG.^7^

Based on a study on knowledge and acceptance of clinical guidelines in clinical practice among general practitioners in Slovenia, adequate knowledge of the guidelines was found in 36.8% of the total study population. The results reveal that knowledge of the guidelines is influenced by the physicians’ acceptance of the guideline recommendations.^8^

With regard to the utilisation of the NATG among the different health care professionals who participated in the current study, the results reveal the most frequent use among pharmacists (38.1%) compared to the least frequent use by nurses (21.7%). Doctors constitute the highest proportion (59.4%) of health care professionals who rarely use the NATG. Laboratory scientists constitute the highest proportion (56.5%) of health care professionals who have never utilised the NATG. The findings of this study reveal that the frequency of the utilisation of the NATG differs among the various health care professionals, even within the same professional group.

On the utilisation of the NATG among the different study centres, the results indicate that the NHA, which is a tertiary health facility, has the highest proportion (45.8%) of health care professionals who utilise the NATG frequently while WGH, a secondary health facility, has the lowest proportion (5.5%). Conversely, UATH and WGA top the chart with the highest proportion of participants who have never utilised the NATG:

12 (44.4%) and nine (50%) respectively. Such findings show a significant variation in the level of utilisation of the NATG among the different treatment centres in Abuja. Similarly, a study conducted in Canada reported that there were huge differences between work settings with regard to clinical guideline utilisation among the 899 decision makers from Canadian provincial health ministries, regional health authorities and hospitals.^9^

For the current study, the results do not show a clear pattern that is linked with this observation, as the UATH, which is also a tertiary site, has a very low percentage of health care professionals who do not utilise the NATG frequently (7.4%). It also has a high percentage of health care professionals who have never utilised the NATG (44.4%).

### Limitations of the study

The study was a quantitative descriptive survey in which close-ended questions constituted the major part of the questionnaires; the barriers were listed by the investigator. It is likely that some barriers faced by some of the participants were not listed or reported, though provision was made for additional responses under ‘others’.

Due to the nature of the study design, causal relationship could not be examined. Some participants who met the inclusion criteria were not available to participate in the study during the period of data collection. The authors would like to acknowledge the limitation posed by the lack of a qualitative component in eliciting the reasons for non-adherence to the NATG by participants in this study. Among the strengths of the study is the fact that all ARV treatment facilities in Nigeria were included in the study and this should result in relatively high data quality.

### Recommendations

Based on the findings of this study, the following recommendations will be useful for the policy makers at the Federal Ministry of Health, Abuja, the National Agency for Control of AIDS, donor agencies, management of hospitals, programme managers and other related bodies.

To ensure proper dissemination of the NATG among health care professionals working in ART centres, all relevant health care professionals should be given free copies of the NATG. The findings of this study show that only 14.4% of the participants have individual copies of the guidelines. Training programmes in ART or HIV/AIDS management targeted at health care professionals should include a module on NATG utilisation.

Periodic continuing professional development programmes should be organised for health care professionals with the aim of providing useful updates on these guidelines.

A follow-up study incorporating a qualitative aspect regarding the non-utilisation of the NATG by health care professionals should be carried out.

### Conclusion

The researchers have noted that the majority of the previous studies focused on measuring the level of adherence to treatment guidelines; however, accessibility of these guidelines could also influence the level of adherence by health care professionals.

This study revealed a gap in the dissemination of the NATG in Abuja among the core health care professionals who provide ART services. As a result of the finding that confirmed the varying levels of utilisation of the NATG among health care professionals working at the ART centres in Abuja, it has become clear that promoting awareness of the existence of the guidelines is important before health care professionals can be assessed on utilisation and other areas of interest related to the guidelines.
